# Supplementary material for: HIVprotI: an integrated web based platform for prediction and design of HIV proteins inhibitors
Source: J Cheminform. 2018 Mar 9;10:12. doi: 10.1186/s13321-018-0266-y (PMC5845081; doi:10.1186/s13321-018-0266-y)
Supplement: Supplementary file 2 — Additional file 2. Source code of HIVProtI web server. [file 13321_2018_266_MOESM2_ESM.zip › HIVprotI_Source-code/hivprotI/weka-3-6-6/doc/weka/classifiers/rules/package-summary.html]

weka.classifiers.rules


---


|  |  |  |
| --- | --- | --- |
| **Overview** \| **Package** \| Class \| **Tree** \| **Deprecated** \| **Index** \| **Help** \| **Weka's home** | |  |
| **PREV PACKAGE**   **NEXT PACKAGE** | **FRAMES**    **NO FRAMES**     **All Classes** |  |


---

## Package weka.classifiers.rules

| **Class Summary** | |
| --- | --- |
| **ConjunctiveRule** | This class implements a single conjunctive rule learner that can predict for numeric and nominal class labels.    A rule consists of antecedents "AND"ed together and the consequent (class value) for the classification/regression. |
| **DecisionTable** | Class for building and using a simple decision table majority classifier.    For more information see:     Ron Kohavi: The Power of Decision Tables. |
| **DecisionTableHashKey** | Class providing hash table keys for DecisionTable |
| **DTNB** | Class for building and using a decision table/naive bayes hybrid classifier. |
| **JRip** | This class implements a propositional rule learner, Repeated Incremental Pruning to Produce Error Reduction (RIPPER), which was proposed by William W. |
| **M5Rules** | Generates a decision list for regression problems using separate-and-conquer. |
| **NNge** | Nearest-neighbor-like algorithm using non-nested generalized exemplars (which are hyperrectangles that can be viewed as if-then rules). |
| **OneR** | Class for building and using a 1R classifier; in other words, uses the minimum-error attribute for prediction, discretizing numeric attributes. |
| **PART** | Class for generating a PART decision list. |
| **Prism** | Class for building and using a PRISM rule set for classification. |
| **Ridor** | An implementation of a RIpple-DOwn Rule learner.    It generates a default rule first and then the exceptions for the default rule with the least (weighted) error rate. |
| **Rule** | Abstract class of generic rule |
| **RuleStats** | This class implements the statistics functions used in the propositional rule learner, from the simpler ones like count of true/false positive/negatives, filter data based on the ruleset, etc. |
| **ZeroR** | Class for building and using a 0-R classifier. |

---


|  |  |  |
| --- | --- | --- |
| **Overview** \| **Package** \| Class \| **Tree** \| **Deprecated** \| **Index** \| **Help** \| **Weka's home** | |  |
| **PREV PACKAGE**   **NEXT PACKAGE** | **FRAMES**    **NO FRAMES**     **All Classes** |  |


---
